# Developing, evaluating and validating a scoring rubric for written case reports

**DOI:** 10.5116/ijme.52c6.d7ef

**Published:** 2014-02-01

**Authors:** Peggy R. Cyr, Kahsi A. Smith, India L. Broyles, Christina T. Holt

**Affiliations:** 1 Department of Family Medicine, Maine Medical Center, Portland, Maine, USA; 2 Center for Outcomes Research and Evaluation, Maine Medical Center, Portland, Maine, USA; 3 Department of Medical Education, University of New England, College of Osteopathic Medicine, Biddeford, Maine, USA

**Keywords:** Scoring rubric, medical student evaluation

## Abstract

**Objectives:**

The purpose of this study was to evaluate Family Medicine Clerkship student**s’** writing skills using an anchored scoring rubric. In this study, we report on the assessment of a current scoring rubric (SR) used to grade written case description papers (CDP) for medical students, describe the development of a revised SR with examination of scoring consistency among faculty raters, and report on feedback from students regarding SR revisions and written CDP.

**Methods:**

Five faculty members scored a total of eighty-three written CDP using both the Original SR (OSR) and the Revised SR1 (RSR1) during the 2009-2010 academic years.

**Results:**

Overall increased faculty inter-rater reliability was obtained using the RSR1. Additionally, this subset analysis revealed that the five faculty using the Revised SR2 (RSR2) had a high measure of inter-rater reliability on their scoring of this subset of papers (as measured by intra-class correlation (ICC) with ICC = 0.93, p < 0.001.

**Conclusions:**

Findings from this research have implications for medical education, by highlighting the importance of the assessment and development of reliable evaluation tools for medical student writing projects.

## Introduction

Writing skills are essential to the practicing physician; therefore, assessment of writing projects by medical students can provide an opportunity to hone critical professional skills. The ability to write clearly and efficiently is critical to performing many essential skills required of physicians such as diagnostic reasoning, management of cases, and overall communication with colleagues and patients. Medical schools may be working from an assumption that writing skills are obtained prior to entering medical school. These skills are rarely taught in a formal capacity during medical school, but are well assessed by an evaluation of a written practical assignment.^1^ Research has shown that when writing skills are taught within medical schools, students demonstrate improved knowledge and overall performance.^2^ The purpose of this study was to evaluate Family Medicine (FM) Clerkship students’ writing skills using an anchored scoring rubric. Various arenas for assessment of FM Clerkship Students’ performance have been delineated, such as clinical skills, communication and writing skills. The Alliance for Clinical Education’s Guide for Clerkship Directors lists sixteen different methods used for clinical education evaluation (ACE Handbook).^3^^,^^4^ One particular evaluation method that is used widely across disciplines is the written case description paper. This assignment requires students to select a topic relevant to family medicine and write a detailed case description of approximately five pages.^5^Evaluation of writing samples requires subjective assessment of a complex performance; therefore, formalized scoring formats are often used (“Scoring Rubric”, SR). A rubric outlines a set of criteria and standards linked to specific learning objectives and may assign a numeric value to coincide with each criteria category. The likelihood that independent evaluators will consistently assign a similar numeric score to the same piece of written work is increased by using anchored descriptors. Scoring rubrics provide the student with feedback outlining the extent to which criteria have been reached, indicating specific areas in which to improve their performance.^6^ Others have developed and studied the use of four point Likert-scale assessment for written case reports.^7^ The use of a scoring rubric allows for a standardized evaluation of performance to enhance consistency when grading subjective assignments and provides essential written feedback to students.As review of previous literature shows, there is not a common practice for consistent evaluation of medical student writing. Therefore, we aimed to develop a tool so that students would have consistent evaluation even when faculty members who evaluate student performance are located in different hospitals in several states. Under these circumstances, it is imperative to use a well devised tool to obtain consistency between various faculty members who are scoring assignments. Furthermore, the process of assessing and revising assessment tools can be an opportunity to educate faculty about the goals of the assignment, and the goals of assessment.^8^^,^^9^The overall goal of this project was to develop a more efficient tool for faculty scoring and to optimize feedback provided to students on their written case description papers. First, we evaluated the current scoring rubric used to score students’ written case description papers in a family medicine third year clerkship. Second, we developed a revised scoring rubric and tested for scoring consistency among faculty using both the old and new version of the rubric. Finally, we obtained qualitative feedback from students regarding rubric revisions and overall course evaluation. Using a process outlined by Green and colleagues,^8^ we focused both on the development of the tool itself and on the process of creating and validating a scoring rubric in our setting.

## Methods

### Design

This study used a mixed methods design employing both quantitative and qualitative data collection methods.

### Participants

This study took place among third year clerkship students in a major medical school in New England, which has two main clerkship sites.

### Sampling method

This study used a convenience sample of third year medical students enrolled in our academic program. There were no exclusion criteria. One third of the students are in the satellite program. All students participate in a Family Medicine clerkship, and ten percent of their grade is based on a written assignment. This scoring rubric study focused on the case description papers written for the rotation, by all of the students. A total of 83 papers were submitted.

### Data collection

All faculty members scored papers using the written scoring rubrics. For the student interviews, one faculty member recorded answers to open ended questions. Survey monkey was used to survey students who were unable to be interviewed in person. Data was sent to the principle investigator (PI) for analysis and storage. This study was sent to both institutions’ Institutional Review Boards where it was exempted from review. Informed consent was waived per the IRB’s approval.

#### Developing the Revised Scoring Rubric (RSR1)

The study began with an evaluation of the scoring rubric that was being used to evaluate students’ written case description papers (Original Scoring Rubric, OSR). The OSR included seven criteria: organization/clarity, focused discussion of key points, knowledge of topic, relevance of topic to family medicine, psycho-social determinants of health, appropriate references to literature, and awareness of how cost influences care. The OSR was rated on a five-point Likert scale anchored with logic and sequencing words. On the OSR, a student could receive 35 possible points. There was also a section at the end of the rubric for faculty to provide narrative comments.Based on the initial review of this current scoring form, the PI devised a Revised Scoring Rubric Version 1 (RSR1) which shifted from Likert scoring to an anchored rubric with keyword descriptors. The categories were renamed to be more descriptive, but kept the basic spectrum of evaluative topics: writing conventions, depth of knowledge/focus, logical sequencing, and topic relevance, biopsychosocial determinants of health, references, and cost issues. After the RSR1 was developed, faculty members from both sites used both rubrics (OSR and RSR1) to score their students’ written case description papers during the 2009-2010 academic years. Each student’s paper was evaluated by two faculty members from their respective site.

#### Revised Scoring Rubric Development

The Revised Scoring Rubric 2 (RSR2) was developed through feedback via a teleconference held to discuss the strengths and weaknesses of the RSR1. The revised draft was subject to further comments and revisions prior to re-implementation. The RSR2 was used to examine scoring consistency and inter-rater reliability among all five faculty members on a subset of seven papers. The seven papers were a purposeful sample selected by the PI to represent higher and lower scoring papers using the OSR. The purpose for selecting papers of both higher and lower quality was to establish benchmarks for scoring various types of papers and to assess the RSR2 ability to differentiate at both ends of the scoring range. All papers were de-identified and randomly distributed to all faculty members for rescoring using the RSR2.

#### Final revisions and feedback

All five faculty members met in person to discuss the results from the revision (RSR2) and to finalize the scoring rubric. Detailed discussions of discrepancies among scoring within each subcategory revealed additional changes to be made. Final changes were incorporated and the Final Revised Version (FRV) was approved by all faculty members.

#### Timeline

The process for evaluating the present scoring rubric, developing and piloting the new scoring rubric (RSR1), revising the scoring rubric (RSR2) and finalizing the format with feedback from student participants took place over one academic year.

#### Qualitative evaluation

Lastly, qualitative evaluation was obtained from medical students at one site. Students were offered the option to provide feedback via individual in-person interview, telephone interview or an online survey. A research team member (not the PI) conducted five individual interviews, four students participated by telephone interview, and three students provided feedback via an online survey. All students were asked for their input regarding their knowledge of how they were being evaluated, the appropriateness of the scoring rubric categories, whether or not they had reviewed the evaluation tools for the written case report and if the scoring rubric added to their understanding of the assignment. These questions were answered on a Likert scale of 1-5. The students were also asked open-ended questions to ascertain which parts of the paper were the hardest to write, suggestions for additional categories and scoring of the rubric as well as any suggestions to make the assignment more interesting for them.

### Data analysis

All quantitative data analysis was performed using the Statistical Package for Social Sciences (SPSS) version 16.0. Consistency among faculty scoring was examined by comparing scores given to students by each faculty member on the same paper using two different scoring rubrics (OSR and RSR1). The closer the scores are to each other indicates higher consistency between raters. The measure of this closeness of score is called inter-rater reliability (IRR) and is assessed by calculating intraclass correlation coefficients (ICC), as described in Kenny et al.^10^ The higher the ICC, the higher the inter-rater reliability among pairs, or, the more likely that the score one trained rater will give would be the same as any other similarly trained rater.Consistency in the RSR2 was examined using the same methods by comparing overall scores and scores from each subcategory for each of seven papers. Range of overall scores and individual category scores were computed to examine for discrepancies among faculty scores. Inter-rater reliability among all five faculty scores was assessed by calculating ICC. Answers to open-ended questions in the qualitative analysis were analyzed thematically and for focused areas of concern.

## Results

Eighty-three student papers were graded by one of three faculty pairs using each of the two scoring rubrics, the OSR and RSR1. Quantitative results are reported for each faculty pair in Table 1. Each student score is reported as the average for the OSR and the RSR1 between the two faculty raters, and the faculty ICC was calculated and shown in Table 1. This table shows that pairs of faculty raters had a lower ICC using the OSR.

**Table 1:** Inter-Rater Reliability Measured as Intraclass Correlation Coefficients (ICC) of Faculty Pairs Using Both the Original Scoring Rubric (OSR) and the Revised Scoring Rubric (RSR1)

ICC Group	Category	Average Measure ICC	95% CI Lower	95%CI Upper	p-value
Pair 1	OSR Total Score	0.79	0.55	0.9	0.001*
N=33	OSR Organization	0.6	0.19	0.8	0.05*
	OSR Focused	0.42	-0.19	0.71	0.069
	OSR Knowledge	0.2	-0.63	0.6	0.27
	OSR Relevance	-0.21	-1.07	0.5	0.523
	OSR Biopsychosocial	-0.1	-1.23	0.46	0.605
	OSR References	0.64	0.27	0.82	0.005*
	OSR Cost	0.79	0.57	0.9	0.001*
	RSR1 Total Score	0.87	0.74	0.94	0.001*
	RSR1 Writing	0.64	0.27	0.82	0.005*
	RSR1 Depth	0.62	0.23	0.81	0.005*
	RSR1 Logical	0.44	-0.14	0.72	0.054
	RSR1 Relevance	0.39	-0.25	0.7	0.087
	RSR1 Biopsychosocial	0.45	-0.12	0.73	0.05*
	RSR1 References	0.64	0.27	0.82	0.005*
	RSR1 Cost	0.79	0.56	0.89	0.001*
ICC Group	Category	Average Measure ICC	95% CI Lower	95%CI Upper	p-value
Pair 2	OSR Total Score	0.38	-0.35	0.71	0.112
N=29	OSR Organization	0.65	0.25	0.84	0.005*
	OSR Focused	0.07	-1.01	0.57	0.426
	OSR Knowledge	0.64	0.22	0.83	0.005
	OSR Relevance	0.8	0.57	0.91	0.001
	OSR Biopsychosocial	0.6	0.14	0.82	0.05
	OSR References	0.24	-0.65	0.65	0.242
	OSR Cost	0.5	-0.08	0.77	0.05*
	RSR1 Total Score	0.64	0.23	0.83	0.005*
	RSR1 Writing	0.73	0.42	0.88	0.001*
	RSR1 Depth	0.23	-0.66	0.64	0.251
	RSR1 Logical	0.32	-0.47	0.69	0.159
	RSR1 Relevance	0.7	0.34	0.86	0.001*
	RSR1 Biopsychosocial	0.4	-0.29	0.72	0.093
	RSR1 References	0.45	-0.19	0.75	0.062
	RSR1 Cost	0.13	-0.88	0.6	0.358
ICC Group	Category	Average Measure ICC	95% CI Lower	95%CI Upper	p-value
Pair 3	OSR Total Score	0.9	0.77	0.96	0.001*
N=21	OSR Organization	0.79	0.48	0.92	0.001*
	OSR Focused	0.8	0.51	0.92	0.001*
	OSR Knowledge	0.9	0.75	0.96	0.001*
	OSR Relevance	0	-1.46	0.59	0.5
	OSR Biopsychosocial	0.68	0.22	0.87	0.05*
	OSR References	0.41	-0.44	0.76	0.12
	OSR Cost	0.76	0.41	0.9	0.001*
	RSR1 Total Score	0.89	0.74	0.96	0.001*
	RSR1 Writing	0.42	-0.43	0.76	0.116
	RSR1 Depth	0.84	0.6	0.93	0.001*
	RSR1 Logical	0.91	0.77	0.96	0.001*
	RSR1 Relevance	0.5	-0.23	0.8	0.065
	RSR1 Biopsychosocial	0.8	0.52	0.92	0.001*
	RSR1 References	0.65	0.13	0.86	0.01*
	RSR1 Cost	0.76	0.41	0.9	0.001*

*p-value < 0.05 determined to be statistically significant

### 

#### Qualitative analysis on the RSR1

The qualitative analysis on the RSR1 from all five faculty members indicated areas of inconsistent interpretations of the scoring criteria. Qualitative descriptions of these areas of discrepancy were clarified through individual and group discussions using thematic analysis. Adjustments were made to the rubric after each discussion and sent to the group by email for a further refinement and verification. This led to the development of the second version of the scoring rubric (RSR2). The faculty approved the new structure and format allowing for more objective scoring. Table 2 shows the general themes which arose from these discussions, and some of the solutions to these thematic issues were included in the anchors. Additions and subtractions to the RSR1 were made after the faculty met to review strengths and limitations.

**Table 2:** General Themes arising from qualitative evaluation of Revised Scoring Rubric (RSR1) and Modifications implemented in Revised Scoring Rubric 2 (RSR2)

Theme area	Representative comment	Modifications
General writing and editing skills	“I can’t assess their grammar and writing skills”	Specific criteria for accuracy and editing standards
Increased specificity in the anchoring descriptions	“This category is simply too vague for me.” “I’m not sure where we indicate if there is medical misinformation in the paper.”	All anchors are more specific
Clarity on the range of acceptable topics for the case descriptions	“I’m confused. A student should be able to write a paper that gets top scores on a basic topic. I don’t want them to write about zebras. This is only a three page paper!” *“We are still seeing a mix. We need to tell the students the recommendation for more complex cases*.”	Choice of acceptable topics more clearly delineated
Overlapping assessment categories for the medical content evaluation	“These categories overlap way too much. They are assessing essentially the same information. The most relevant aspects of these two categories are: was there a discussion and summary of the information and an in- depth analysis of the differential diagnosis.”	Categories clarified and condensed
Focus on the behavioral aspects of description	“I know what this means because I have been a practicing family doctor for years, but I am not sure how to explain it to students.”	Clear anchors in a specific behavioral medicine category were developed
Understanding the nature of the references (classic cases vs. relevant references)	“I don’t want to just look at the number of references, but do the references support the conclusion of the paper? This is more important”. “Some references are old, but still landmark articles, so the student should not be penalized for an old reference”.	Clarity about relevance, timing and impact of references incorporated

#### RSR1 Revision results (RSR2)

Seven papers were selected and scored by all five faculty members using only the RSR2. There was consistency among scores for higher quality papers (mean score = 19.05, SD = 2.52) and lower quality papers (mean score = 12.73, SD = 3.57) out of a possible 21 points. Results revealed significant ICC between all five faculty raters’ overall scores for each paper and in each subcategory on all seven papers using the RSR2 (Table 3). This shows that the RSR2 allows consistency in scoring for trained faculty reviewers.

**Table 3:** Inter-Rater Reliability Measured as Intraclass Correlation Coefficients (ICC) of All Faculty Raters Using the Revised Scoring Rubric (RSR2)

ICC Group	Category	Average Measure ICC	95% CI Lower	95% CI Upper	p
5 Faculty Raters	RSR2 Total Score	0.93	0.79	0.99	0.001*
N=7	RSR2 Writing	0.89	0.67	0.98	0.001*
	RSR2 Depth	0.85	0.54	0.97	0.001*
	RSR2 Logical	0.82	0.46	0.97	0.001*
	RSR2 Relevance	0.71	0.12	0.94	0.05*
	RSR2 Biopsychosocial	0.69	0.06	0.94	0.05*
	RSR2 References	0.94	0.81	0.99	0.001*
	RSR2 Cost	0.89	0.66	0.98	0.001*

*p-value < 0.05 determined to be statistically significant

#### Final revisions of RSR2 (FRV)

After the RSR2 was piloted, a faculty meeting was held to discuss the results. Concerns about specific criteria and form and content of the RSR2 were raised. Further refinements were included, and a Final Revised Version (FRV) created (Appendix A). The new rubric was approved for use on both campuses with new rotations starting March 2010.

#### Student feedback

Twelve of the satellite site students supplied feedback via survey questions conducted after the course. Thematic analysis revealed that a majority of students strongly agreed that they were aware of how they were being evaluated on their case description papers (7/12, 58.3%) and had looked at the evaluation tools while writing their papers (6/12, 50%). Students also strongly agreed that the scoring rubric categories seemed appropriate and added to their understanding of their assignment (9/12, 75%).In open ended questions, students indicated concerns about the following three categories: Biopsychosocial Determinants of Health, Cost Issues, and References. Students considered the bio-psychosocial category to be too broad and identified this category as the hardest to write. This issue was addressed through revision of the rubric to include actual descriptors of biopsychosocial aspects of a case - family, living situation, impact of disease on life, perspective on their illness, and state of psychological health (see Appendix A). Regarding Cost Issues, students recommended a change in the instructions for this category, which was addressed by including a clear definition of cost issues with specific examples in the FRV. Finally, several students reported difficulty with understanding what types of references were required. The final revision of this category includes clarification of the requirement for use of current literature from the last five years and evidence that the references support the conclusions of the paper. At least one student indicated interest in having top papers submitted for publication.

## Discussion and conclusions

Our analysis of the OSR indicated a need to improve reliability and develop more useful descriptors for students’ work. Our process of revising the scoring rubric allowed greater internal consistency and reliability as well as improved guidance for students. Finally, our analysis of student feedback yielded additional insights into how students interpret assignments and scoring systems. Future work will need to use independent faculty who had never scored papers to assess the ease of use of the RSR2 tool.

### Implications

Results from this study highlight the importance of clearly defined anchoring criteria in scoring rubrics in order to ensure consistency among scorers. Furthermore, revising and testing scoring rubrics by content experts is a labor-intensive process, involving multiple phases, resulting in a more reliable tool. This is not a novel idea regarding written work, since studies have shown inter-rater reliability can be achieved when experienced evaluators meet regularly to refine criteria.^11^Although our curriculum does not include teaching about writing skills, the scoring rubric and written instructions can set the standard for the improving quality of written work by medical students. Posting exemplar papers for students to access and read serves as an additional resource without requiring formal didactic instruction. The student feedback provided important ideas for the implementation of the assignment with more concise written instructions. Students and faculty identified the Bio-psychosocial Determinants of Health and Cost Issues categories as most difficult to write and most difficult to score, respectively.Medical students are continuously being assessed in multiple realms of performance. The ongoing evaluation of our assessment tools should become common practice in Family Medicine Clerkships. This study highlights the importance of developing clear criteria for scoring rubrics in evaluating medical student writing. These well-defined scoring rubrics assist the student in completion of complex performance assignments. Assessment of inter-rater reliability of scoring among faculty strengthens the internal consistency of the tool. Scoring rubrics should be evaluated and validated by expert faculty and the medical student users. These efforts will lead to a more robust tool. Having faculty and students work collaboratively enhances our medical student education.

### Limitations

There are a few limitations to this study that merit discussion. First, the initial revision to the scoring rubric was done solely by the principal investigator. Making the initial revisions as a joint effort of multiple experts may have resulted in a superior tool. This was also a single medical school study with a small convenience sample which limits generalizability. Student feedback would have been strengthened with information collected before and after the change in the scoring rubric. Nevertheless, our approach served as a valuable faculty development process, and a student curriculum development process.

## 

### Conflict of Interest

The authors declare that they have no conflict of interest.

## Appendix A

### Scoring Rubric: Final Revised Version (FRV)

#### Family Medicine Clinical Core Clerkship

Case Description Paper Evaluation (Total Point Available = 70)

NAME: DATE: TOPIC:

**Table:**

Criteria	Inadequate (6 points each)	Needs Improvement (8 points each)	Meets Expectations (9 points each)	Exceeds Expectations (10 points each)
Writing Conventions	Poor organization and sentence structure impedes comprehension. Errors distract the reader.	Needs editing, several grammati-cal, punctuation and/or format-ting errors.	Only 2-3 minor errors in grammar, punctuation, and/or formatting.	Writing style and grammar are very high quality. Minimal editing needed in punctuation or formatting.
Logical Sequencing	Completely unorganized case presentation.	2-3 errors in sequencing or 2-3 sections missing.	1 error in sequencing or 1 section missing.	No errors in sequencing. All relevant information is present.
Topic Relevance	Topic is uncommon and not translated into relevant Family Medicine care issues.	Common presentation, but focus is not relevant to Family Medicine care issues.	Common presentation and focus adequately translated into Family Medicine care issues.	Common presentation and translates expertly into Family Medicine care issues.
Depth of Knowledge	No analysis or discussion of differential diagnosis. Incorrect medical infor-mation.	Brief analysis and limited discussion of differential diagnosis.	Topic well developed and adequate analysis of differential diagnosis.	Excellent discussion and in depth analysis of differential diagnosis.
Biopsychosocial Determinants of Health	Errors in and/or no descriptors that make this patient unique. Paper is culturally insensitive.	Describes 1-2 psychosocial aspects that make this patient unique.	Describes 3-4 psychosocial aspects that make this patient unique.	Describes 5 psychosocial aspects that make this patient unique (family, living situation, impact of disease on life, perspective on their illness, and state of psychological health).
Cost Issues	No description of how cost influences medical decision making and patient impact.	Cost issues described, but incomplete awareness of how cost influences medical decision-making and impact on the patient.	Full discussion of cost issues and well linked to medical decision-making and impact on the patient.	Captures nuances of how cost influences medical decision making and impact on the patient.
References	No references in the paper.	References are not relevant and do not support the conclusions of the paper.	3-4 references, majority are within the last 5 years and adequately support the conclusions of the paper.	5 or more references with the majority the last 5 years, including a review article, which expertly support the conclusions of the paper.
